# Effects of morphology and pore size of mesoporous silicas on the efficiency of an immobilized enzyme†

**DOI:** 10.1039/d1ra01358k

**Published:** 2021-03-08

**Authors:** Ping-Chung Kuo, Zhi-Xun Lin, Tzi-Yi Wu, Chun-Han Hsu, Hong-Ping Lin, Tian-Shung Wu

**Affiliations:** a School of Pharmacy, College of Medicine, National Cheng Kung University Tainan 701 Taiwan tswu@mail.ncku.edu.tw +886-6-2740552 +886-6-2747538; b Department of Chemistry, National Cheng Kung University Tainan 701 Taiwan hplin@mail.ncku.edu.tw +886-6-2757575 ext. 65342; c Department of Chemical & Materials Engineering, National Yunlin University of Science and Technology Yunlin 644 Taiwan; d General Education Center, National Tainan Junior College of Nursing Tainan 700 Taiwan; e Department of Pharmacy, College of Pharmacy and Health Care, Tajen University Pingtung 907 Taiwan

## Abstract

An investigation is performed into the efficiency of the *Streptomyces griseus* HUT 6037 enzyme immobilized in three different mesoporous silicas, namely mesoporous silica film, mesocellular foam, and rod-like SBA-15. It is shown that for all three supports, the pH value changes the surface charge and charge density and hence determines the maximum loading capacity of the enzyme. The products of the enzyme hydrolytic reaction are analyzed by ^1^H-NMR. The results show that among the three silica supports, the mesoporous silica film (with a channel length in the range of 60–100 nm) maximizes the accessibility of the immobilized enzyme. The loading capacity of the enzyme is up to 95% at pH 7 and the activity of the immobilized enzyme is maintained for more than 15 days when using a silica film support. The order of the activity of the enzyme immobilized in different mesoporous silica supports is: mesoporous silica film > mesocellular foam > rod-like SBA-15. Furthermore, the immobilized enzyme can be easily separated from the reaction solution *via* simple filtration or centrifugation methods and re-used for hydrolytic reaction as required.

## Abstract 2

Mesoporous silica films were used as supports with high loading capacity and enzyme activity.

## Graphical abstract

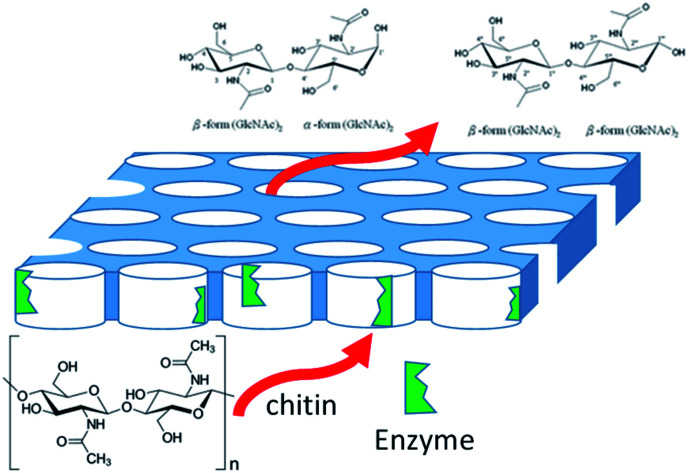

## 1. Introduction

Silica-based materials have been extensively studied in various fields of chemistry and materials science recently, with particular emphasis on their application as antibacterial agents,^1^ biosensors,^2^ supports, and adsorbents.^3,4^ Silica-based materials include, but are not limited to, porous glass,^5^ sol–gel silica,^6^ and mesoporous materials.^4,5^ Among these materials, mesoporous silicas (MSs) have attracted particular attention as a platform for the protection of enzyme molecules due to their large, well-ordered and rigid pore structures.^7^ Various MSs have been synthesized and utilized for bio-immobilization purposes, including MCM-41,^8^ SBA-15,^9^ and mesocellular foam (MCF).^10^

The pore sizes of MSs are tunable in the range of 2–50 nm and are thus comparable to the dimensions of typical enzymes (typically 3–7 nm). As a result, they have attracted great interest as matrix materials for enzyme immobilization.^11–23^ Various studies have shown that MCM-41 materials have a pore size of just 3.0 nm, and are thus limited to the immobilization of enzymes with small sizes.^15–17^ Furthermore, despite their high surface areas (*ca.* 1000 m^2^ g^−1^), they allow only relatively low enzyme loadings (typically <10 wt%) and slow enzyme immobilization rates.^18–20^ As a result, recent studies on enzyme loading have focused more on SBA-15 materials with pore sizes of *ca.* 5–15 nm (ref. 21) and mesocellular siliceous foams with pore sizes of 15–40 nm.^22^ Many studies have investigated the enzymatic activity and stability of MS platforms.^23–28^ In general, the results have shown that adsorption, covalent bonding and entrapment mechanisms all provide an effective means of stabilizing enzymes on inorganic, organic or polymeric matrices by increasing the rigidity of the matrix and reducing the unfolding and deactivating possibility.^29–32^

Diaz and Mansor examined the MS entrapment of three different enzymes, namely cytochrome C, papain and trypsin^33,34^ It was shown that the trypsin retained a significant activity for more than one week in the immobilized state, but was totally deactivated after just 24 h in the free (*i.e.*, solution) state. Wang and Caruso entrapped catalase, peroxidase, cytochrome C, and lysozyme enzymes in MS supports, and showed that all four enzymes exhibited good activity and a long durability.^35,36^ Many studies have shown that enzymes retain high activity when loaded in MS hosts.^34–36^ However, due to the relatively small pore size of MSs, the kinetics of the encapsulated enzymes are reduced.^37–39^ Consequently, the enzyme catalytic reaction time is prolonged. Furthermore, un-immobilized enzymes tend to stick to the unreacted reactant and dissolve in the solution after the reaction process. Hence, recovering the enzyme from the reaction solution is time consuming and expensive.

In the present study, *Streptomyces griseus* HUT 6037 (*S. g*-HUT 6037) enzyme is immobilized on three different MSs, namely mesoporous silica film, mesocellular foam, and rod-like SBA-15, using a physical adsorption technique. The effects of pH on the enzyme-loading capacity of the three MS platforms are examined and compared. The products of enzyme hydrolytic reaction in the presence of chitin are then analyzed by ^1^H-NMR. Finally, the economic potential of the mesoporous silica film is demonstrated by recovering the enzyme from the reaction solution using a simple centrifugation method and then reusing the recycled enzyme to perform a further hydrolytic reaction process.

## 2. Experimental Section

### 2.1 Materials

Most of the chemicals used in the present study, and *S. g*-HUT 6037 enzyme with a molecular mass of *ca.* 27 kDa (dimensional size ∼2 nm), were purchased from Sigma-Aldrich (St. Louis, MO, USA), and were of reagent grade or higher. Chitin was obtained from Kiotec Co. (Hsinchu, Taiwan). PEG 6000 and PEG 10000 were acquired from Pan Asia Chemical Co. (Taipei, Taiwan).

### 2.2 Preparation of mesoporous silicas

#### 2.2.1 Preparation of mesoporous silica films^40^

Vertical mesoporous silica films were synthesized in a CTAB–SDS–P123–water (0.75 g–0.99 g–25.70 g–125 g) system under pH 4.5 using an SDS/CTAB molar ratio of 1.67, a reaction temperature of 45 °C, and an aging time of 3 minutes. Then, a 2.75 g sodium silicate solution (≥27% SiO_2_ basis, Sigma-Aldrich) with 150 g acidified aqueous solution (containing 5 g 1.2 M H_2_SO_4(aq)_), use NaOH_(aq)_ to adjust the pH to 4.5. After the condensation time of 3 minutes, the above CTAB–SDS–P123–water system was added and produced a white precipitate. The mixture was further placed in stainless steel pressure autoclaves with Teflon containers and aged for 24 h at 100 °C. Finally, the surfactant was removed by calcination at 600 °C for 6 h.

#### 2.2.2 Preparation of mesocellular foams^41^

Mesocellular foams were prepared using a hydrothermal method. Briefly, a pH 5.0 mixture of P123 : toluene : water/sodium silicate : water = 1.4 : 4.0 : 50/5.5 : 300 was stirred at 40 °C for 24 h. The samples were further placed in stainless steel pressure autoclaves with Teflon containers and aged for 24 h at 100 °C. The surfactant was then removed by calcination at 560 °C for 6 h.

#### 2.2.3 Preparation of rod-like SBA-15 materials^42^

Rod-like MSs were prepared by stirring a pH 4.0 mixture of P123 : water/sodium silicate : water = 1.6 : 50/8.75 : 300 at 30 °C for 24 h. The samples were further placed in stainless steel pressure autoclaves with Teflon containers and aged for 24 h at 100 °C. The surfactant was then removed by calcination at 560 °C for 6 h.

### 2.3 Characterization

Thermogravimetric analysis (TGA) was performed on a TA-Q50 thermogravimetric analyzer at a ramp rate of 20 °C min^−1^ in air. Transmission electron microscopy (TEM) images were taken with a Hitachi H-7100 device. For each sample, the Brunauer–Emmett–Teller (BET) specific surface area was obtained from the nitrogen adsorption isotherms measured on a Micromeritics ASAP 2020 system. The pore size distribution was calculated using the BJH method. The ^1^H NMR spectra were recorded on a Bruker Avance 500 NMR spectrometer, with 3-(trimethylsilyl)propionic-2,2,3,3-d4 acid sodium salt (TSP-d4) as an internal standard. The chemical shifts were reported in *δ* values (ppm). The amount of un-immobilized enzyme in the solution was estimated using a UV-vis-NIR measurement system (UV-Vis Hitachi U-4100).

### 2.4 Enzyme immobilization

Enzyme-immobilized MSs were prepared by adding 10.0 mg of the prepared MSs to 1.4 mg *S. g*-HUT 6037 in 1 mL D_2_O phosphoric acid buffer solution (pH 7.0) and allowing immobilization to proceed at 4 °C for 6 h. According to the supplier, the *S. g*-HUT 6037 enzyme had a pI (the isoelectric point) value of 7.1. After removing the buffer solution, the immobilized enzyme was washed serially with two 10 mL portions of distilled water and one 10 mL portion of 0.1 M D_2_O phosphate buffer solution. The immobilized enzyme derivatives were then re-suspended in the same buffer in a cold room until required for use.

### 2.5 Enzyme hydrolytic reaction

10.0 mg chitin was added to the prepared immobilized enzymes in 950 μL D_2_O phosphoric acid buffer, and 50 μL 0.01 M TSP-d_4_ solution was then added as an internal standard. The composition of the resultant mixture after hydrolytic reaction was monitored at 37 °C and 100 rpm in the NMR tube. The products were then analyzed using a ^1^H-NMR spectroscope.

### 2.6 ^1^H-NMR quantitative analysis

Five different concentrations of each sample were prepared and the amounts of the internal standards [(GlcNAc) and (GlcNAc)_2_] (Fig. S1†) were estimated through the integral values of the signals and calibration curves. The calibration curves of (GlcNAc) and (GlcNAc)_2_, respectively, showed good linearity with *R*^2^ values higher than 0.999 in both cases (Fig. S2†). The limits of detection for (GlcNAc) and (GlcNAc)_2_ were determined by measuring the signal-to-noise ratios of serial dilutions.

## 3. Results and discussion

### 3.1 Characterization of mesoporous silica films, mesocellular foams and rod-like SBA-15

**Fig. 1:**
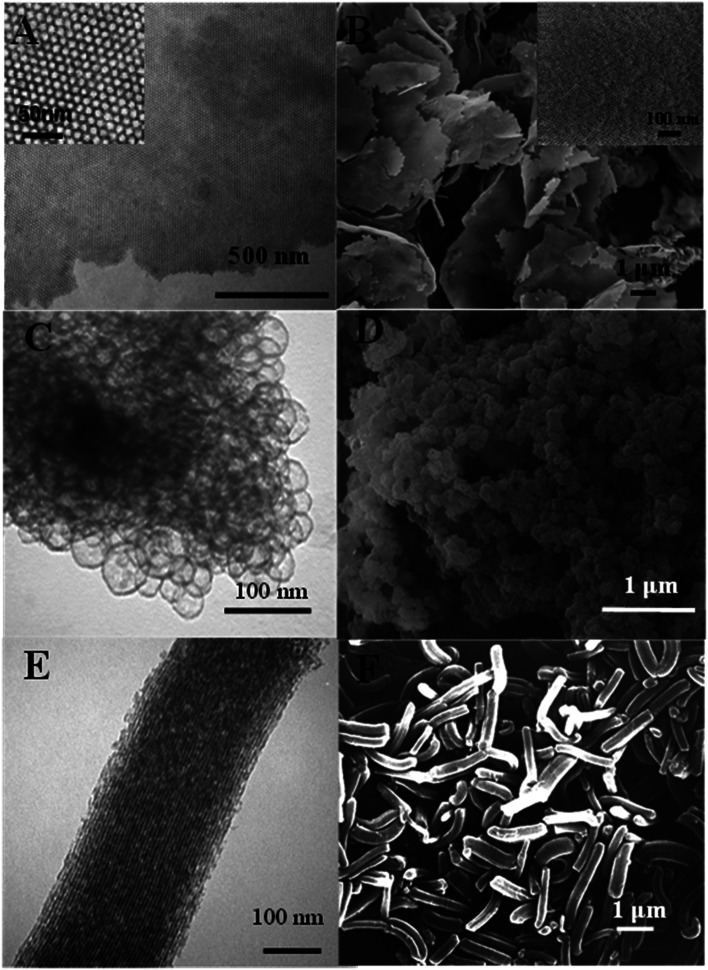
TEM and SEM images of (A and B) mesoporous silica film, (C and D) mesocellular foam, and (E and F) rod-like SBA-15.

**Fig. 2:**
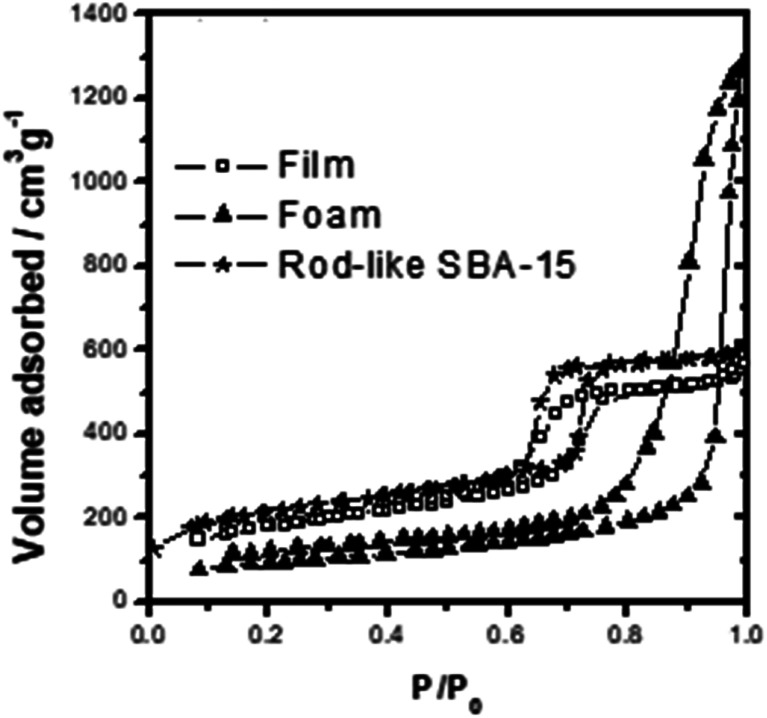
The N_2_ adsorption–desorption diagrams of three type MSs.

The TEM and SEM images in Fig. 1A and B shows that the mesoporous silica film has a well-ordered hexagonal packed (*P*6*mm*) mesostructure. As shown in Fig. 2, the film has a type-IV N_2_ sorption curve, and is hence inferred to be composed of cylindrical nanochannels. Moreover, the analytic results show that the mesoporous film has a BET surface area of *ca.* 400 m^2^ g^−1^, a pore size of approximately 6.0 nm, and a pore volume of 0.83 cm^3^ g^−1^. TEM and SEM images in Fig. 1C and D shows that the mesocellular foam has a hollow vesicle structure with a diameter of 30–60 nm. In addition, according to the N_2_ sorption curve (Fig. 2), the foam has a BET surface area of *ca.* 300 m^2^ g^−1^, a pore size of 26.4 nm, and a pore volume of 1.85 cm^3^ g^−1^. Finally, the TEM and SEM images in Fig. 1E and F shows that the SBA-15 material has a typical rod-like morphology with well-ordered nanochannels and a rod length of *ca.* 2.0 μm. According to Fig. 2, the SBA-15 sample has a surface area of 515 m^2^ g^−1^, a pore size of around 6.1 nm, and a pore volume of 0.82 cm^3^ g^−1^.

### 3.2 Loading capacities of mesoporous silica materials

**Fig. 3:**
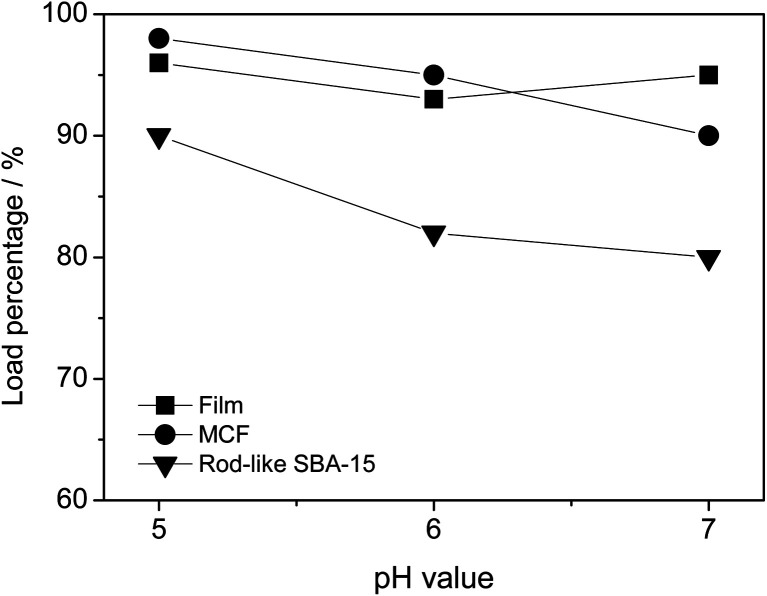
Enzyme loading capacities of different MSs under various pH values.

In general, the –NH and –OH functional groups of *S. g*-HUT 6037 enzyme form hydrogen bonds with the high-density Si–OH (silanol) functional groups on the surface of mesoporous silica materials.^7,42^ Consequently, in the present study, the *S. g*-HUT 6037 enzyme is readily immobilized on all three MS platforms. Fig. 3 shows the enzyme loading capacities of the different MS materials under various pH values, as determined from the corresponding UV-vis spectra (Fig. S3†). For ease of visualization, the loading capacities of the three materials under different pH conditions are also summarized in Table 1. For all three materials, the loading capacity varies with the pH value; with the maximum capacity occurring under pH 5.0 in every case. It is hence inferred that the charge densities of the enzyme and Si–OH functional group on the surface of the MSs change with the pH value. The neutral pH value of the present *S. g*-HUT 6037 enzyme is around 7.1 (see Section 2.4). Thus, at pH 5.0, the surface of the enzyme displays a partially positive charge. Moreover, the order of positive-charge density at different pH values is pH 5.0 > pH 6.0 > pH 7.0. In addition, the surfaces of the MSs possess a negative charge above pH 2.0.^7^ As a result, the interaction of the enzyme and silica materials is enhanced in more acidic environments (*i.e.*, pH 5.0), and hence the loading capacity of the *S. g*-HUT 6037 enzyme increases accordingly.

**Table 1:** The loading capacity (%) of different types MSs at various pH values

	pH 7	pH 6	pH 5
Mesoporous silica film	95%	93%	96%
MCF silica	90%	95%	98%
Rod-like SBA-15	80%	82%	90%

### 3.3 Enzyme hydrolytic reaction and ^1^H-NMR quantitative analysis of chitin hydrolytic enzyme *S. g*-HUT 6037

For enzymes immobilized on a support, the enzyme configuration is fixed and some active sites are sheltered or the structure is partially distorted. As a result, the number of active sites is reduced and hence the activity of the enzyme is degraded. In the present study, the activity and stability of the *S. g*-HUT 6037 enzyme immobilized on the three different MS platforms were investigated by means of chitin hydrolytic reaction and ^1^H-NMR quantitative analysis.

**Fig. 4:**
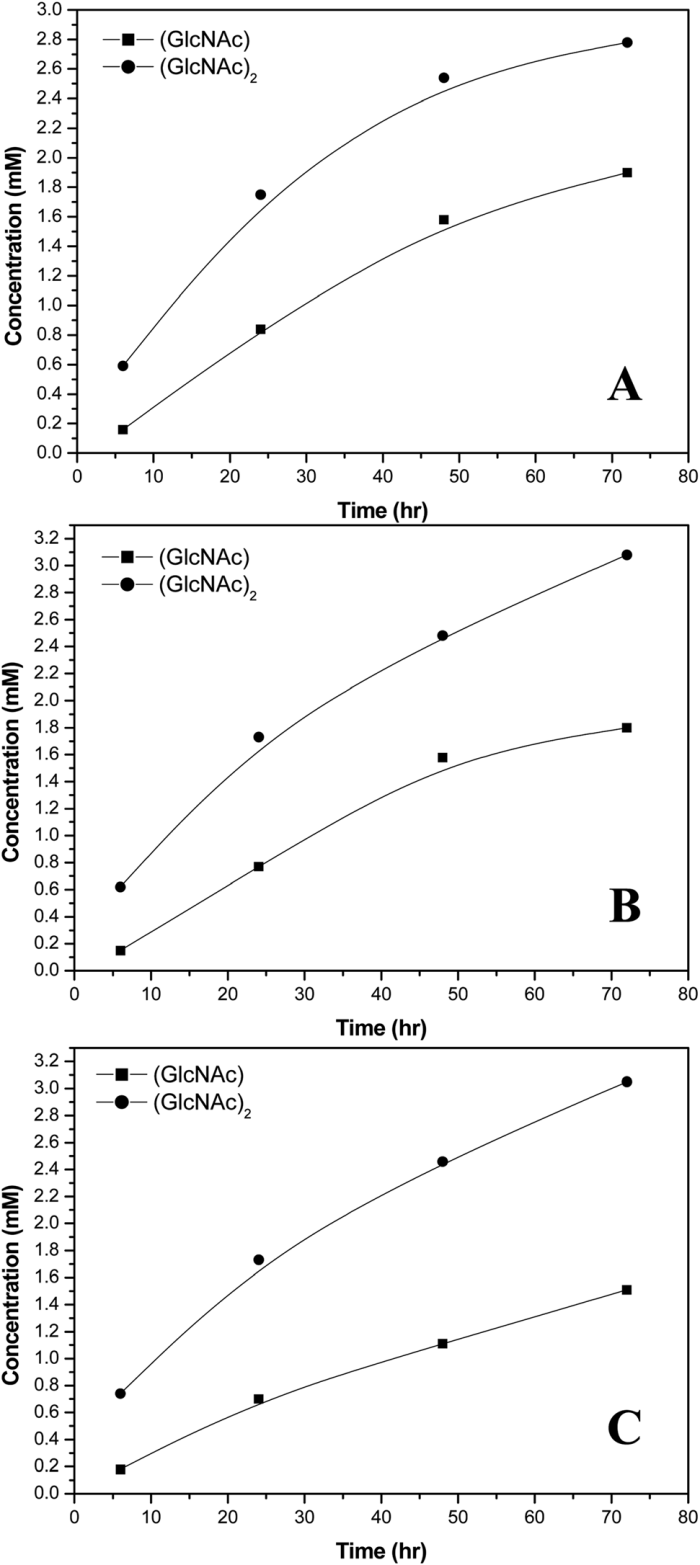
Concentrations of chitin hydrolytic reaction products catalyzed by *S. g*-HUT 6037 in: (A) pH 5.0, (B) pH 6.0, and (C) pH 7.0 environments.

D_2_O phosphoric acid buffer solutions at different pH values were prepared and chitin hydrolytic enzymes were used to implement a hydrolytic reaction. The hydrolytic reactions were terminated at different reaction times and the concentrations of the reaction products were determined *via*^1^H-NMR quantitative analysis. Chitin hydrolytic enzyme *S. g*-HUT 6037 is a type of exonuclease with major products of (GlcNAc)_2_ and GlcNAc (Fig. 4). Table S1† shows the analytic data obtained for various concentrations of (GlcNAc)_2_. As shown in Fig.S2,† the calibration curves for (GlcNAc)_2_ and (GlcNAc) both exhibit good linearity with *R*^2^ values of 0.9990 and 0.9996, respectively. Thus, the accuracy of the ^1^H-NMR detection results is confirmed. It is seen in Fig. S2† that he acetyl signals of GlcNAc and (GlcNAc)_2_ in the ^1^H-NMR spectra are overlapped. Thus, Bruker Deconvolution simulation software (Fig. S4†) was used to simulate the areas of the GlcNAc and (GlcNAc)_2_ signals such that the concentrations of hydrolytic products could be more accurately determined. Since the sample concentrations are less than 0.909 mM (*i.e.*, the detection limit of 500 MHz ^1^H-NMR), the Bruker nosepr1d program pulse method was used to remove the background interference of water and obtain the final ^1^H-NMR signal of the chitin hydrolytic products (Fig. S5†).

Referring to Fig. 4, it is seen that the concentrations of (GlcNAc)_2_ and GlcNAc are highly dependent on the environmental pH value. For example, at pH 7.0, the amount of (GlcNAc)_2_ is greater than that of GlcNAc, while at pH 5.0, the reverse is true. The chitin hydrolytic reaction produces (GlcNAc)_2_ as the main product originally, and as the reaction time increases, the amount of GlcNAc and (GlcNAc)_2_ increases. However, at pH 5.0, the amount of (GlcNAc)_2_ increases more slowly than that of GlcNAc due to the acidic hydrolysis of (GlcNAc)_2_ to GlcNAc in acidic environments. As a result, the relative amount of (GlcNAc)_2_ is lower than that of GlcNAc (Fig. 4A). Comparatively, under neutral conditions (pH 7.0), (GlcNAc)_2_ is not easily hydrolyzed to GlcNAc, and hence the relative amount of (GlcNAc)_2_ is greater than that of GlcNAc (Fig. 4C). It is noted that this finding is of significant practical interest since, of all the hydrolyzed products of chitin, (GlcNAc)_2_ has a higher economic value.

### 3.4 Enzyme immobilization in different mesoporous silicas

#### 3.4.1 Rod-like MS

The use of rod-like MS as a platform for enzyme immobilization has been extensively investigated.^24^ As shown in Table 1, the present rod-like SBA-15 achieves a maximum *S. g*-HUT 6037 loading capacity of 90% under pH 5.0 conditions. Comparing the reaction rates of the non-immobilized enzyme and the SBA-15 immobilized enzyme, respectively (see Table 2 and Fig. S6†), it is seen that the SBA-15 immobilized enzyme preserves a high catalytic activity at pH 6.0, but that of the non-immobilized enzyme reduces dramatically. In other words, it appears that immobilization in the long (>1.0 μm) nanochannels of the SBA-15 mesoporous silica would induce the sheltering or destroying of the active sites in the *S. g*-HUT 6037 enzyme. Referring to Fig. S6,† it is seen that while SBA-15 immobilization protects the *S. g*-HUT 6037 enzyme and maintains its catalytic activity at various pH values for at least 15 days, the overall catalytic activity decreases due to the low accessibility of the long nanochannels.

**Table 2:** The hydrolytic reaction of different type MSs-immobilization enzyme and pure enzyme (none) after 1 day

Conc. (mM)	None		Film-type MS		MCF		SBA-15	
	(GlcNAc)_2_	GlcNAc	(GlcNAc)_2_	GlcNAc	(GlcNAc)_2_	GlcNAc	(GlcNAc)_2_	GlcNAc
pH 5.0	1.76	0.83	2.26	1.41	1.65	0.72	1.26	0.61
pH 6.0	1.73	0.78	1.92	1.07	1.77	0.88	1.15	0.56
pH 7.0	1.71	0.72	1.27	0.52	1.00	0.54	1.46	0.65

#### 3.4.2 Film type MS

**Fig. 5:**
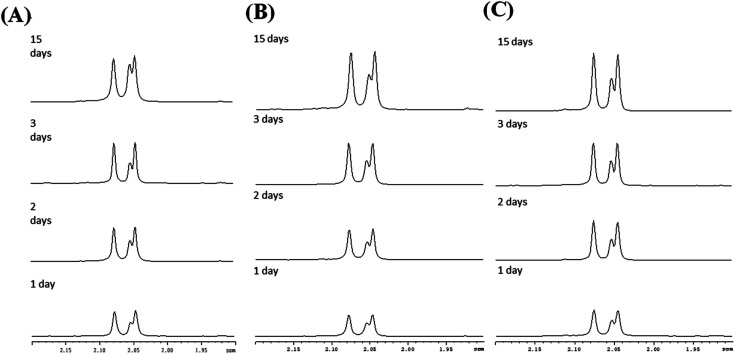
^1^H-NMR spectra of chitin hydrolytic products in: (A) pH 7.0, (B) pH 6.0 and (C) pH 5.0 environments with film type MS.

**Fig. 6:**
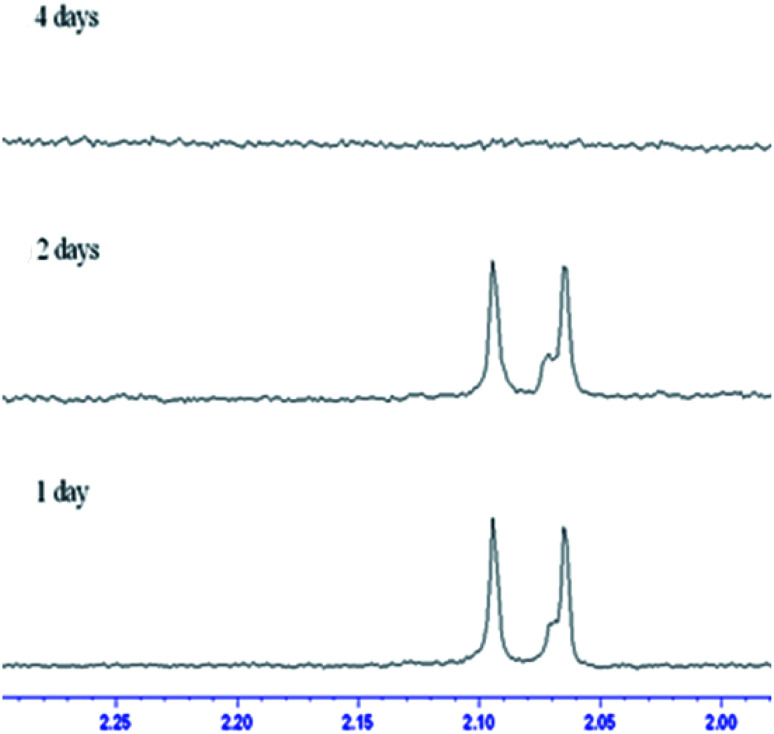
Change in enzyme hydrolytic products over time in H_2_O phosphoric acid buffer solution (pH ≈ 7.0).

In contrast to the rod-like SBA-15 mesoporous silica described above with long nanochannels, the film type MS consists of short nanochannels with a length of just 200 nm.^40–42^ Due to the short diffusion length, the immobilized enzyme in the MS film is highly accessible to the environment and hence the catalytic activity increases. Fig. 5 shows the chitin hydrolytic reactions at pH 5.0, 6.0 and 7.0, respectively, after reaction periods of 1–15 days. It is seen that both (GlcNAc)_2_ and GlcNAc are produced after one day of reaction. As shown in Table 2, the two enzymes exhibit similar catalytic activities. Moreover, the catalytic activities do not decrease significantly under acidic pH conditions. However, under neutral conditions (pH 7.0), the activity of the non-immobilized enzyme is maintained for only 5 to 7 days (Fig. 6). In other words, for reaction times longer than 7 days, the amount of hydrolytic products (GlcNAc and (GlcNAc)_2_) remains almost unchanged. By contrast, the activity of the immobilized enzyme is maintained for more than 15 days. In other words, since the nanochannels in the film-type MS are short, most of the active sites of the immobilized enzymes are accessible to the environment, and consequently the efficiency of the chitin hydrolysis reaction with the film-immobilized enzyme is significantly enhanced.

#### 3.4.3 Mesocellular foam

To understand the effect of the pore size on the accessibility, the MCFs supports with pore size of 26.4 nm can encapsulate the enzymes in nanopores. Comparing the reaction rates of the non-immobilized enzyme and MCF-immobilized enzyme (Table 2 and Fig. S7†), it is found that the MCF-immobilized enzymes retain their activity over time under acidic conditions, but have a lower yield of hydrolytic reaction products than the film-immobilized enzymes. However, at pH 7.0, the catalytic activity of the immobilized enzyme decreases significantly after just one day. This finding is reasonable since the enzyme loading capacity of the MCF matrix is relatively low (*ca.* 90 wt%). Furthermore, the micrometer-sized MCF matrix (a pore size of 26.4 nm) results in a low accessibility of the active sites, and hence the overall catalytic activity is lower than that of the film-type MS. Nonetheless, for all of the considered pH values, the MCF-immobilized enzyme still exhibits catalytic activity for at least 15 days. In other words, MCF appears to be a favorable platform for the long-term immobilization and protection of *S. g*-HUT 6037 enzyme.

### 3.5 Application and development of enzyme immobilization

The large-scale production of enzyme catalytic reaction products is generally not commercially feasible due to the difficulties and expense involved in separating and purifying the enzyme following the reaction process. However, enzymes are expensive and typically available in only limited quantities. Thus, to support industrial applications, it is desirable to develop more effective technologies for recycling enzymes with a high efficiency and low cost.

**Fig. 7:**
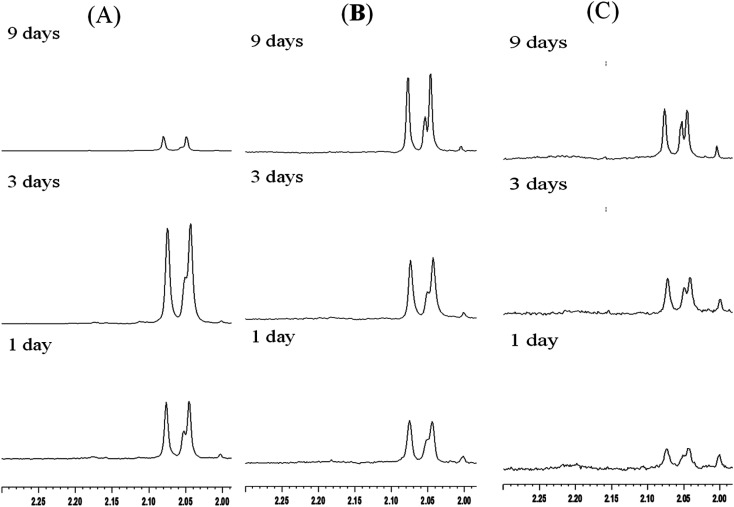
^1^H-NMR spectra of chitin hydrolytic products in: (A) pH 7.0, (B) pH 6.0 and (C) pH 5.0 environments with film type MS and recycled immobilized enzyme.

As described in Section 1 of this paper, the literature contains various protocols for enzyme immobilization.^11–14^ Such methods not only have the advantages of improving the stability and life time of the enzyme, but also enable the separation of the enzyme from the reaction solution *via* a simple centrifugal or filtration process. According, the present study has investigated the use of three different mesoporous silica materials (mesoporous silica film, mesocellular foam, and rod-like SBA-15) as supports for the immobilization of *S. g*-HUT 6037 enzyme. After chitin hydrolytic reaction, non-immobilized enzymes generally adhere to the residual chitin or dissolve in the reaction mixture, and are hence not easily separated from the reaction products, even with centrifugal and filtration methods. By contrast, for the immobilized enzymes considered in the present study, the enzymes are easily recovered *via* centrifugation and can then be reused for further hydrolytic reaction. Fig. 7 shows the ^1^H-NMR spectra obtained for the recycled film-immobilized enzyme. The results are very similar to those shown in Fig. 5 for the original immobilized enzyme, and hence confirm the feasibility for recycling the enzyme following the original hydrolytic reaction.

## 4. Conclusions

This study has investigated the use of three different mesoporous silica supports, namely mesoporous film, mesocellular foam and rod-like SBA-15 for the immobilization of *S. g*-HUT 6037 enzyme. It has been shown that the loading capacity and activity of the immobilized enzyme depend on both the support type and the pH value. Among the three supports, the film-type MS provides the highest activity due to its short nanochannel length, which maximizes the accessibility of the enzyme to the environment. By contrast, the rod-like SBA-15 and mesocellular foam, with a longer channel length and small pore size respectively, render the immobilized enzyme less accessible to the reactant and therefore reduce the activity. Although partial activity loses, the stability of the immobilized enzyme still increases. Notably, all three immobilized enzymes can be recovered from the solution following the reaction process using a simple centrifugation method. Overall, the results show that film-type MS provides a highly promising candidate material for enzyme immobilization and paves the way toward the production of enzyme catalytic reaction products on an industrial scale.

## Conflicts of interest

There are no conflicts to declare.

## Supplementary Material

RA-011-D1RA01358K-s001
