# Supplementary material for: Effects of morphology and pore size of mesoporous silicas on the efficiency of an immobilized enzyme
Source: RSC Adv. 2021 Mar 8;11(17):10010–7. doi: 10.1039/d1ra01358k (PMC8695390; doi:10.1039/d1ra01358k)
Supplement: RA-011-D1RA01358K-s001 [file RA-011-D1RA01358K-s001.pdf]

Electronic Supplementary Material (ESI) for RSC Advances.
This journal is © The Royal Society of Chemistry 2021

## Supporting information

## Effects of Morphology and Pore Size of Mesoporous Silicas on Efficiency of Immobilized Enzyme

Ping-Chung Kuo,[1] Zhi-Xun Lin,[2] Tzi-Yi Wu,[3] Chun-Han Hsu,[4] Hong-Ping Lin,*[,3] Tian-Shung Wu*[,1,5]
[1] School of Pharmacy, College of Medicine, National Cheng Kung University, Tainan 701, Taiwan; [2] Department of Chemistry, National Cheng Kung University, Tainan 701, Taiwan; [3] Department of Chemical & Materials Engineering, National Yunlin University of Science and Technology, Yunlin 644, Taiwan; [4] General Education Center, National Tainan Junior College of Nursing, Tainan 700, Taiwan;[5] Department of Pharmacy, College of Pharmacy and Health Care, Tajen University, Pingtung 907, Taiwan

Table S1. Analytic data of different concentrations of *N,N'*-diacetylchitobiose

| | The integral value at δ 0 (I.S.) | The integral value of $(GlcNAc)_2$ at δ 2.08 and 2.05 | | The actual concentration of $(GlcNAc)_2$ (mM) | $(GlcNAc)_2$ conversion value (mM) |
|---|---|---|---|---|---|
| 1 | 120.491 | 4.968 | 4.819 | 0.909 | 0.99705 |
| 2 | 103.170 | 10.524 | 10.65 | 0.681 | 0.75850 |
| 3 | 110.806 | 20.455 | 20.791 | 0.4545 | 0.50759 |
| 4 | 100.272 | 27.589 | 27.258 | 0.227 | 0.27987 |
| 5 | 115.023 | 40.753 | 41.764 | 0.0909 | 0.11076 |

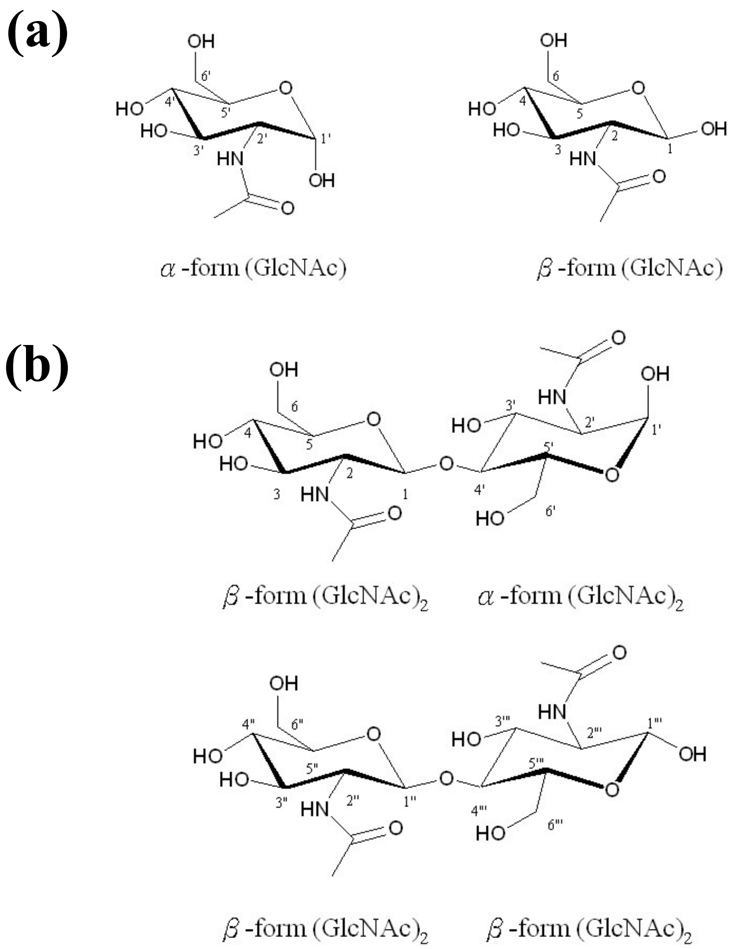


Figure S1. Chemical structures of: (a) GlcNAc and (b) $(GlcNAc)_2$.

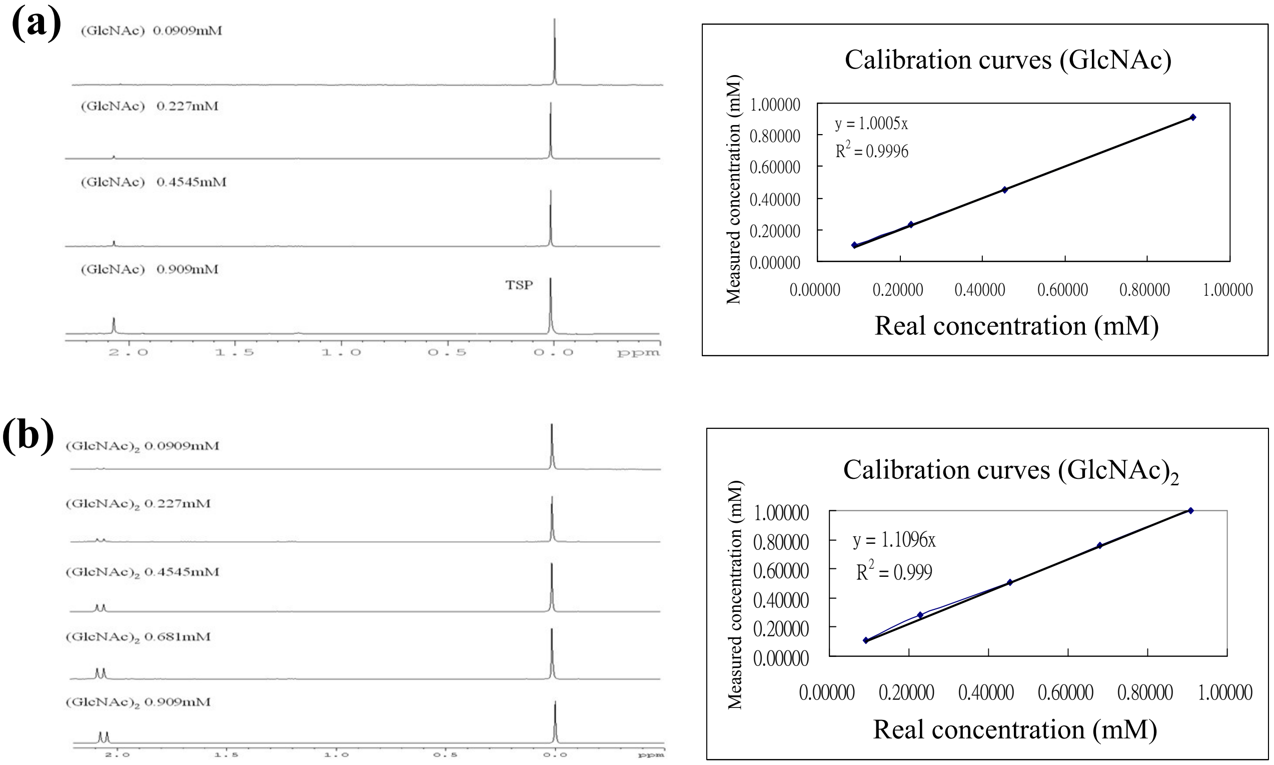


Figure S2. $^1$H-NMR quantitative analysis and calibration curves of: (a) (GlcNAc) and (b) $(GlcNAc)_2$ (I.S：Tsp-$d_4$).

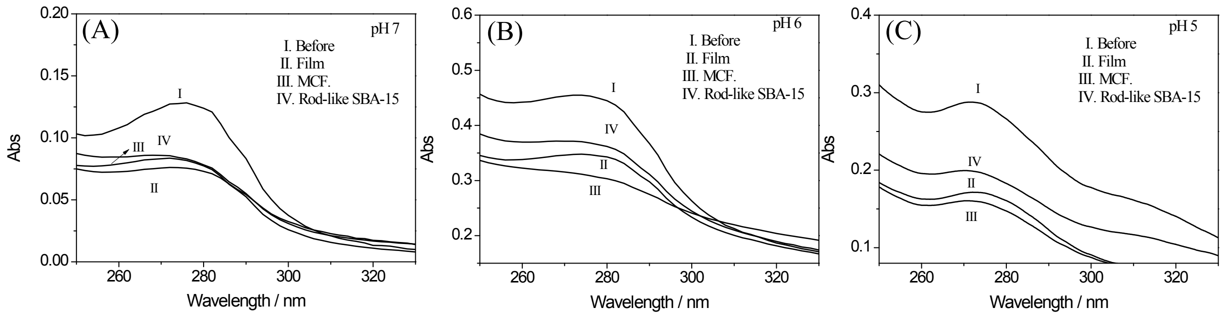


Figure S3. UV-vis spectra of enzyme before and after loading in three different MSs under different pH values.

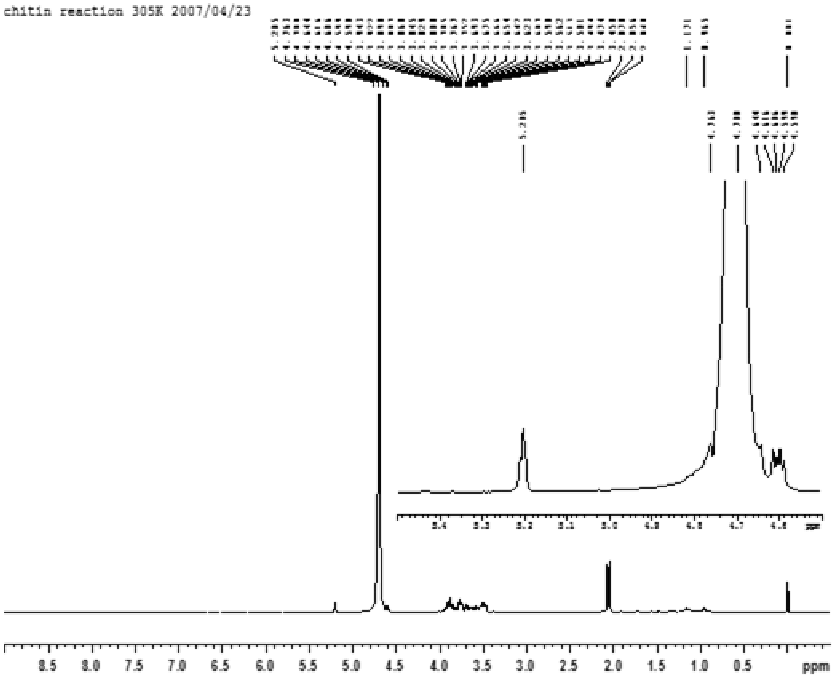


Figure S4. $^{1}$H-NMR signal of chitin hydrolytic product (without nosepr1d program).

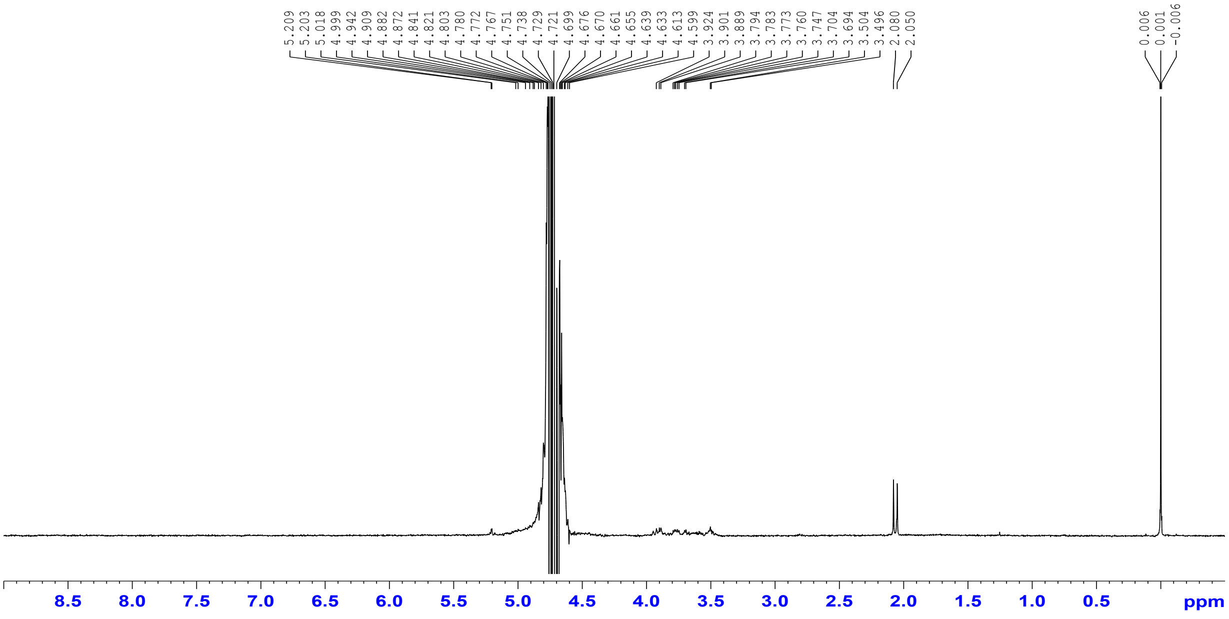


Figure S5. $^{1}$H-NMR signal of chitin hydrolytic product (with nosepr1d program).

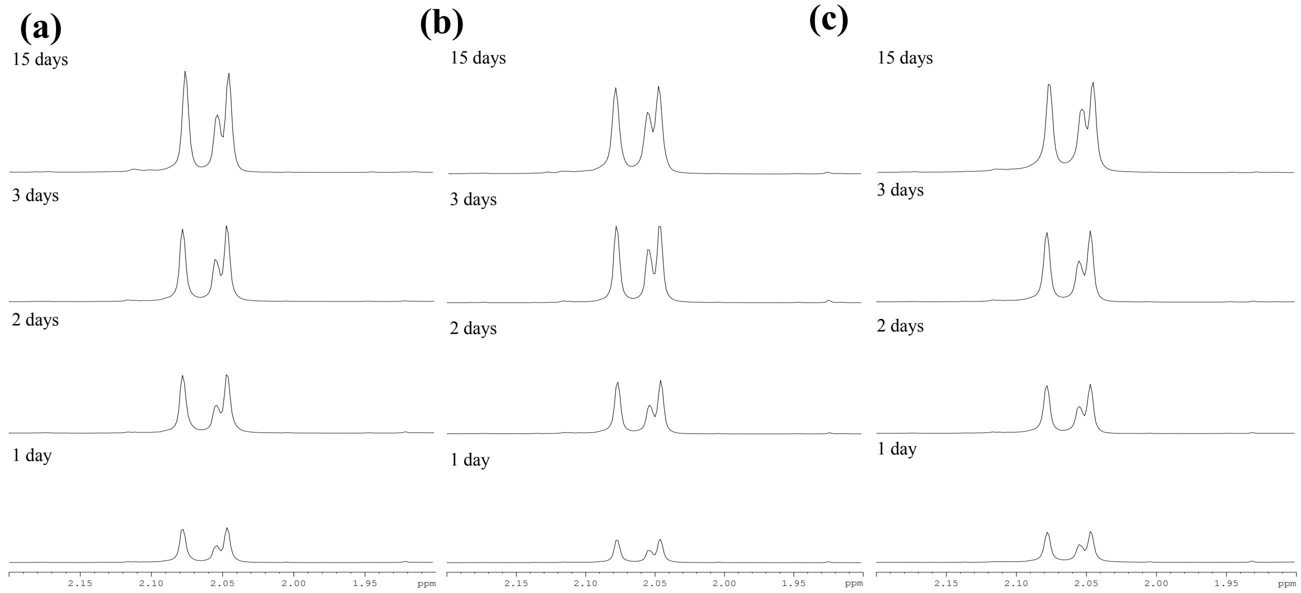


Figure S6. $^1$H-NMR spectra of chitin hydrolytic reactions in: (a) pH 7.0, (b) pH 6.0 and (c) pH 5.0 environments with rod-like SBA-15.

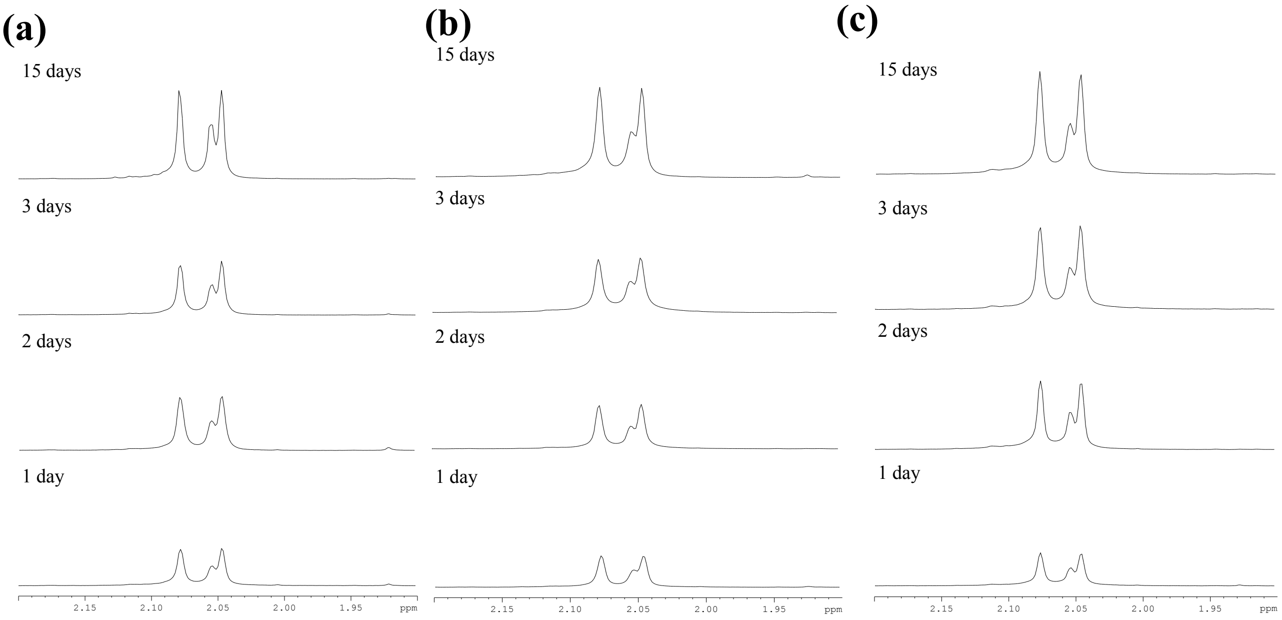


Figure S7. $^1$H-NMR spectra of chitin hydrolytic reactions in: (a) pH 7.0, (b) pH 6.0 and (c) pH 5.0 environments with MCF.
